# Antioxidant Enzymes and Heat-Shock Protein Genes of Green Peach Aphid (*Myzus persicae*) Under Short-Time Heat Stress

**DOI:** 10.3389/fphys.2021.805509

**Published:** 2021-12-17

**Authors:** Aroosa Khurshid, Rehan Inayat, Ansa Tamkeen, Inzamam Ul Haq, Chunchun Li, Solomon Boamah, Jing-Jiang Zhou, Changzhong Liu

**Affiliations:** ^1^College of Plant Protection, Gansu Agricultural University, Lanzhou, China; ^2^Department of Entomology, Faculty of Agriculture, University of Poonch, Rawalakot, Pakistan; ^3^State Key Laboratory of Green Pesticide and Agricultural Bioengineering, Ministry of Education, Guizhou University, Guiyang, China

**Keywords:** green peach aphid, *Myzus persicae*, heat stress, heat-shock protein, antioxidant enzymes

## Abstract

The management of insect pests under fluctuating temperatures has become an interesting area of study due to their ability to stimulate defense mechanisms against heat stress. Therefore, understanding insect’s physiological and molecular response to heat stress is of paramount importance for pest management. Aphids are ectothermic organisms capable of surviving in different climatic conditions. This study aimed to determine the effects of short-time heat stress on green peach aphid *Myzus persicae* under controlled conditions. In this study, short-time heat stress treatments at different temperatures 27, 30, 33, and 36°C with exposure times of 1, 3, 6, and 10 h, respectively, on the activities of antioxidant enzymes, including superoxide dismutase (SOD), catalase (CAT), peroxidase (POD), and oxidants, such as malondialdehyde (MDA) and hydrogen peroxide (H_2_O_2_), were determined. The results showed that the short-time heat stress significantly increased the content of MDA of *M. persicae* by 71, 78, 81, and 86% at 36°C for the exposure times of 1, 3, 6, and 10 h, respectively, compared with control. The content of H_2_O_2_ increased by 75, 80, 85, and 88% at 36°C for the exposure times of 1, 3, 6, and 10 h, respectively, compared with the control. The SOD, POD, and CAT activities increased by 61, 76, and 77% for 1 h, 72, 83, and 84% for 3 h, 80, 85, and 86% for 6 h, and 87, 87.6, and 88% for 10 h at 36°C, respectively, compared with control. Again, under short-time heat stress, the transcription levels of *Hsp22*, *Hsp23*, *Hsp27*, *SOD*, *POD*, and *CAT* genes were upregulated compared with control. Our results suggest that *M. persicae* increased the enzymatic antioxidant activity and heat-shock gene expression as one of the defensive mechanisms in response to heat stresses.

## Introduction

*Myzus persicae* (Sulzer) (Homoptera: Aphididae), also known as the green peach aphid, is a highly adaptable and polyphagous insect pest that feeds on more than 400 plant species from 40 plant families, including many economically important crops (Kennedy et al., 1962; Stevens and Lacomme, 2017). Aphids are used as a model insect in studies of the effects of climate change on insect biology because they develop over a small temperature range, and their growth rate is highly dependent on temperature (Hulle et al., 2010; Durak et al., 2016).

Temperature fluctuations affect aphid morphology and metabolism, which can lead to cell damage. The defense responses of aphids to high temperatures are of great interest. Aphids can suffer from physiological and ecological stress due to heat stress (Dong et al., 2013; Sentis et al., 2015). The upper temperature threshold for the growth of populations of many aphid species has been estimated to be between 25 and 30°C, while the lower temperature threshold has been estimated to be below 5°C (Barlow, 1962). Optimal temperatures for reproduction were 25°C for *M. persicae* and 20°C for *Macrosiphum euphorbiae* (Barlow, 1962). Acclimation temperature affected heat coma; this relationship was linear for *Myzus ornatus* and *Myzus polaris* but non-linear for *M. persicae* (increased tolerance at 10 and 25°C). Upper critical temperatures in *M. persicae* range from 38.5°C (Broadbent and Hollings, 1951) to 42°C (Hazell et al., 2010). Previous studies indicate that *M. persicae* has the greatest tolerance to high temperatures (Hazell et al., 2010). Dampc et al. (2020) also focused on three temperatures, i.e., 20, 25, and 28°C, and showed that the adaptive mechanisms were activated by the flexible activity of enzymes, which ran more efficiently at higher temperatures. The defense responses of *Aphis pomi* varied as a function of temperature at 28°C and survived due to flexible enzyme activity.

There is a close relationship between insect responses to heat and many heat-shock proteins (HSPs), which act as chaperones to aid protein production and refolding after stress and may also enhance immune responses (Wojda, 2017). Numerous studies on the interaction between insects and thermal stress have shown that insects have evolved complex protective mechanisms to protect themselves from high temperatures. HSPs and antioxidant enzymes have been the most prominent effectors in this process (Yang et al., 2010; King and MacRae, 2015). The short forms of heat shock, such as Hsp22, Hsp23, and Hsp27, and mRNAs are approximately 100 nucleotides longer than their respective hormone-induced short forms in *Drosophila* cells. Expression of the small HSP genes can be induced in cultured *Drosophila* cells by high-temperature shock and exposure to physiological doses of the insect molting hormone, ecdysterone (Morganelli et al., 1985). The Hsp110 genes function in an ATP-dependent manner as a nucleotide exchange factor that releases peptide substrate from Hsp70. They respond differently to heat stress in different organisms within the same genus, and their expression can be caused by heat stress in animals, according to a recent review (Jones et al., 2018). The enzymatic defense response of aphids is reflected in changes in the activity of enzymatic markers in their tissues (Dampc et al., 2020). As stress proteins, HSPs are involved in the protection of proteins under oxidative and hypertonic stress induced by extreme temperatures, UV radiation, xenobiotic exposure, and parasitoid infestation (Kang et al., 2017). Therefore, knowing the transcription level of HSPs in aphids under different heat stress situations is beneficial for future agricultural management.

In general, the production of reactive oxygen species (ROS) and antioxidant scavenging processes is in equilibrium. Heat stress, in contrast, disturbs this equilibrium and leads to increased production of ROS, resulting in lipid peroxidation (LPO) through the destruction of cell lipids (Dubovskiy et al., 2008; Lalouette et al., 2011; Schieber and Chandel, 2014). The major antioxidant enzymes in insects are superoxide dismutase (SOD), catalase (CAT), and peroxidase (POD) (Felton and Summers, 1995; Wang et al., 2001). A significant increase in antioxidant enzyme activities indicates oxidative stress and a sign of a good ability to counteract oxidative stress by removing ROS from cells (Jia et al., 2011; Zhang et al., 2015). Previous work has been done on the antioxidant enzyme activity of *M. persicae* with different host plants. There have also been changes in antioxidant enzyme function in *M. persicae*, a highly polyphagous genus, due to its tolerance to different host plants (Abdelsalam et al., 2016), but to the best of our knowledge, no studies have been conducted to calculate the combined effect of different temperatures and time intervals on antioxidant enzyme activity and gene expression of *M. persicae*. Therefore, this research aimed to observe how temperature changes affect the activities of antioxidant enzymes and HSPs genes in response to short-time heat stress in the green peach aphid. This type of research will lead to new insights into the molecular processes underlying heat resistance in *M. persicae*, as well as new opportunities to promote the use of heat treatments to control this pest.

## Materials and Methods

### Insects and Host Plant

Adult green peach aphids (*M. persicae*) were collected from the potato experimental farm of Gansu Agricultural University, Lanzhou, China. The aphids were kept under controlled conditions in a climate chamber and reared in the laboratory on potato seedlings under the photo exposure time of 16:8 h light/dark, 60 ± 5% humidity, and 24 ± 1°C. The adults from the third generation were used for each experiment. The host plant potato (*Solanum tuberosum* L.) (*Solanaceae*) tubers were obtained from Gansu Seeds Research Laboratory, China, planted in pots and watered regularly.

### Heat Stress Treatment

Adult aphids from the third generation were treated with four different temperatures, i.e., 27, 30, 33, and 36°C, in a climatic chamber under the constant humidity of 60 ± 5% and the photo exposure time of 16:8 h light/dark (Ma and Ma, 2012) developed a new parameter, drop-off temperature (DOT), to describe the critical temperature at which an aphid drops off its host plant when the ambient temperature rises, finding that adults starved for 12 h had higher DOT values than those who were unstarved or starved for 6 h and that behavioral thermoregulation and energy acquisition were in competition. Hence, the heat stress exposure time of this study was chosen under 1, 3, 6, and 10 h. The treatments were achieved by using a programmable temperature controller. Each combinatory treatment of temperature and exposure time was repeated three times with 30 aphids each time. Briefly, 30 aphids were transferred to a 9 cm Petri dish using a camel hair brush and supplied with potato leaves, moist filter paper, and a water-moistened cotton ball for hydration. The aphids were then immediately transferred from 25°C (control temperature) to the target temperatures at 27, 30, 33, and 36°C at different times 1, 3, 6, and 10 h. The aphids from the original colony at 25°C were used as an untreated control group for each combined treatment of temperature and exposure time. All treated aphids were collected and frozen in liquid nitrogen and stored at −80°C for analysis.

### Determination of Reactive Oxygen Species Scavenging Enzyme Activity

Aphids (30 individuals) from each treatment were homogenized using an electric mechanical homogenizer in a phosphate buffer (0.1 M, pH 7.0) at 0°C. The homogenate was centrifuged at 8,000 × *g* for 10 min at 4°C. The supernatant was used to assay the activity of SOD (EC 1.15.1.1), POD (EC 1.11.1.7), and CAT (EC 1.11.1.6) with a spectrophotometer (SP-756P, Shanghai, China), and enzyme activities were expressed as units mg per fresh weight of aphids (μ mg^–1^ FW). All enzyme activity was measured at a controlled temperature of 25°C. The SOD activity was measured at 560 nm (Wang et al., 2001). The aphid homogenate 100 μl was mixed with 500 μl of 0.4 mM nitroblue tetrazolium in phosphate buffer 0.2 M (pH 7.8) and xanthine solution (0.25 mM). The mixture was incubated for 20 min, and then the absorbance was measured at 560 nm (TECAN Infinite 200 microplate reader). The POD activity was determined at 470 nm (Fehrmann and Dimond, 1967). The aphid homogenate 100 μl was mixed with 0.1 M phosphate buffer (pH 7.0), distilled water 20 μl, and 0.2 M pyrogallol, and incubated at 30°C for 25 min. Then, 50 μl of 25% trichloroacetic acid (TCA) solution was added. The absorbance was taken with the TECAN Infinite 200 microplate reader at a wavelength of 470 nm. The CAT activity was measured and assayed according to the method described by Aebi (1984) with minor modifications. We added 30 mM H_2_O_2_ to the aphid homogenate, and the disappearance of H_2_O_2_ was measured at 240 nm with the spectrophotometer.

### Measurement of Malondialdehyde and H_2_O_2_ Contents

The content of malondialdehyde (MDA) was determined according to the method described by Buege and Aust (1978) with some modifications. The samples of treated and untreated aphids (0.1 g) were homogenized in 100 μl of 5% (w/v) TCA and centrifuged at 10,000 × *g* for 5 min. The supernatant (0.5 ml) was mixed with 1 ml of 0.5% (w/v) thiobarbituric acid in 20% TCA and used for the MDA assay. The absorbance of each sample was measured at 532 nm and corrected for non-specific turbidity by subtracting the absorbance at 600 nm. Following the protocol of Mostofa and Fujita (2013) with minor modification, the hydrogen peroxide (H_2_O_2_) content was measured. Briefly, frozen aphids samples (0.1 g) were homogenized using an electric mechanical homogenizer and centrifuged at 12,000 × *g* for 20 min, and the supernatant was collected and reacted with titanium tetrachloride (TiCl_4_) and ammonium hydroxide. After the second centrifuge, the supernatant was discarded, and the precipitate was washed recurrently with cold acetone until it turned colorless. The washed precipitate was dissolved in 20 ml of 2 M MH_2_SO_4_, and the absorbance was measured at 415 nm against a blank. The standard H_2_O_2_, which was prepared with 100 μl of the 0.1 mM H_2_O_2_ standard in 900 L distilled water to make a 10 μl H_2_O_2_ standard, was treated with TiCl_4_ and subjected to a similar process.

### Expression of Heat-Shock Proteins and Antioxidant Enzyme Genes in *Myzus persicae*

Total RNAs were extracted from 100 mg treated and untreated aphids using the total RNA extraction kit (Solarbio Biotechnology, Beijing, China). The quantity of RNA was determined using a Nano-Drop spectrophotometer at the absorbance of 230 and 260 nm. The purity of the RNA was verified by the A260/A230 ratio and 1% agarose gel electrophoresis (Supplementary Figure 1). According to the protocol of the manufacturer, the cDNA was synthesized using the M5 Hiper RT cDNA Synthesis Kit (Mei5 Biotechnology, Beijing, China). The gene-specific primers for the *Hsp22* (NCBI gene ID: 3772576), *Hsp23* (NCBI gene ID: 39077), *Hsp27* (NCBI gene ID: 43901), *SOD* (NCBI gene ID: 111035379), *POD* (NCBI gene ID: 112683716), and *CAT* (NCBI gene ID: 111041019) genes and the internal control *actin gene* (NCBI gene ID: 836110) were designed using the Primer Express 3.0 software based on the sequences of target genes in NCBI, and are listed in Supplementary Table 1.

The quantitative real-time PCR was conducted in 20 μl reaction mixture containing 2× M5 Hiper Real-Time PCR Super-Mix (10 μl), primer (1 μl), sample cDNA (1 μl), and sterilized ultrapure grade H_2_O (8 μl). The PCR amplifications were performed with the following cycling conditions: at 95°C (10 min), followed by 40 cycles of denaturation at 95°C for 15 s, annealing at 60°C for 30 s, and extension at 72°C for 30 s. The relative expression level was quantified using the comparative 2^–ΔΔCt^ method (Livak and Schmittgen, 2001). Each gene was analyzed three times for each of the three biologically independent treatments.

### Statistical Analysis

The interactions between two main effects of temperatures and stress exposure times were analyzed with a two-way analysis of variance (ANOVA). The significant interactions were identified. The average enzymatic activities and gene expression were then analyzed using one-way ANOVA and compared between treatments. The data were analyzed using ANOVA in SPSS version 16.0 (SPSS Inc., Chicago, IL, United States), and mean comparisons were made using Tukey’s HSD test (*p* < 0.05).

## Results

### Antioxidant Enzyme Activities

**FIGURE 1:**
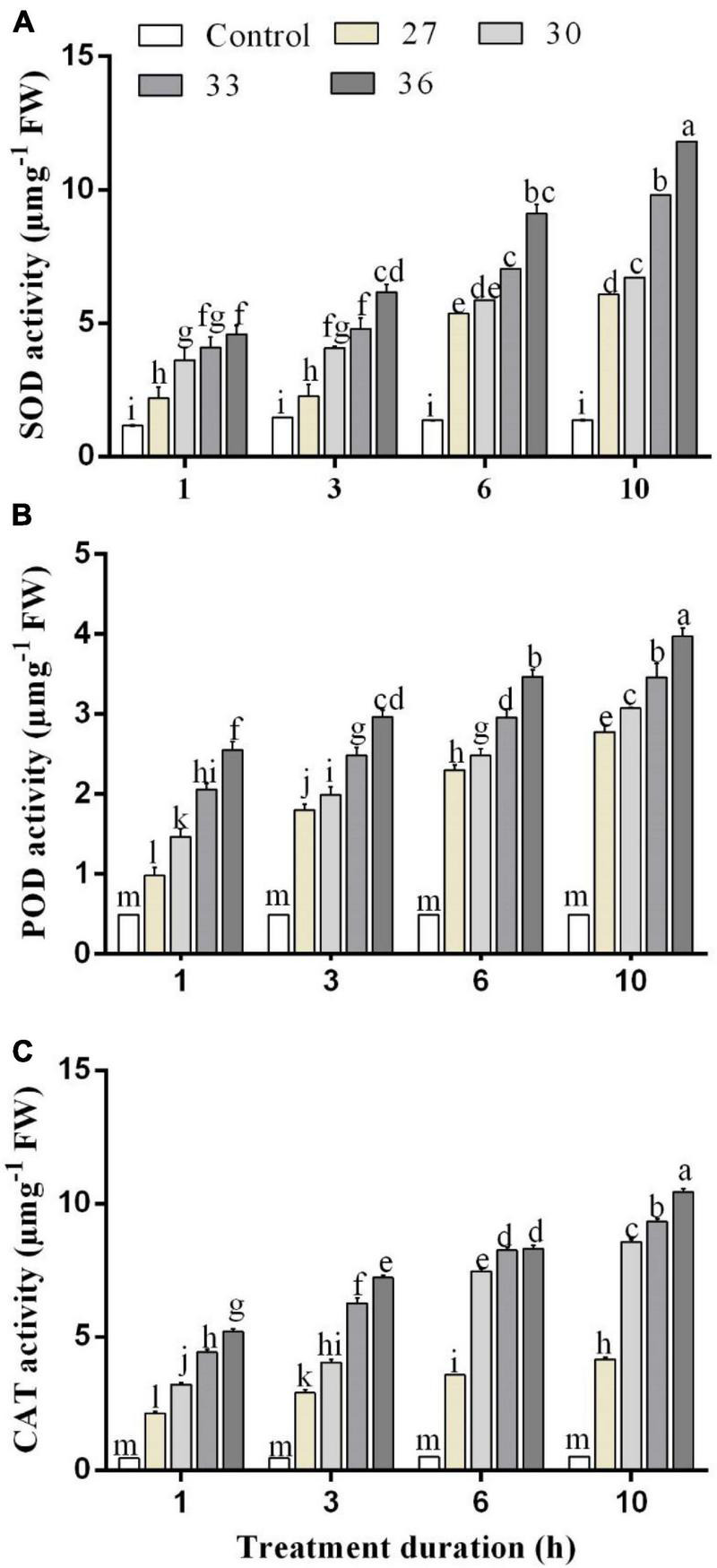
**(A)** Superoxide dismutase (SOD), **(B)** peroxidase (POD), and **(C)** catalase (CAT) activity in *M. persicae* under different heat stresses (27, 30, 33, and 36°C) at different durations (1, 3, 6, and 10 h) in growth chambers. Small bars represent the standard errors of the means. Different lower case letters indicate significant differences between treatments at Tukey’s HSD test (*p* < 0.05) using one-way ANOVA. FW is the fresh weight of aphids.

The changes in the SOD activity of *M. persicae* after the heat shock at different temperatures and over different exposure times are shown in Figure 1A. The SOD activity increased significantly at all temperatures (*p* < 0.001) and durations (*p* < 0.001), compared with control (25°C), and there was a significant interaction between temperature and durations (*p* < 0.001). The SOD activity increased as the temperature increased at different temperature treatments of 27, 30, and 33°C, respectively, for 1 h (2.2 ± 0.3, 3.6 ± 0.4, and 4.1 ± 0.4 μ mg^–1^ FW), 3 h (2.3 ± 0.3, 4.1 ± 0.1, and 4.8 ± 0.3 μ mg^–1^ FW), 6 h (5.4 ± 0.2, 5.9 ± 0.1, and 7.0 ± 0.2 μ mg^–1^ FW), and 10 h (6.1 ± 0.2, 6.7 ± 0.2, and 9.8 ± 0.3 μ mg^–1^ FW) as compared with the control at 25°C (1.1 ± 0.1, 1.4 ± 0.1, 1.3 ± 0.0, and 1.3 ± 0.1 μ mg^–1^ FW). The highest activity was found at 36°C (4.6 ± 1.0 μ mg^–1^ FW for 1 h, 6.2 ± 0.5 μ mg^–1^ FW for 3 h, 9.1 ± 0.3 μ mg^–1^ FW for 6 h, and 11.8 ± 0.2 μ mg^–1^ FW for 10 h) and increased by 61, 72, 80, and 87% for 1, 3, 6, and 10 h, respectively, compared with the control treatment.

The POD activities of *M. persicae* under different heat stress are shown in Figure 1B. The POD activity in *M. persicae* adults was also significantly increased at all temperatures (*p* < 0.001) and all durations in comparison with control and temperature and duration interacted significantly (*p* < 0.001). The highest activities were found for exposure times of 1, 3, 6, and 10 h at 36°C (2.4 ± 0.3 μ mg^–1^ FW for 1 h, 2.9 ± 0.4 μ mg^–1^ FW for 3 h, 3.4 ± 0.2 μ mg^–1^ FW for 6 h, and 4.1 ± 0.4 μ mg^–1^ FW for 10 h). It increased in various degrees for 1 h exposure time by 50.2% at 27°C, 66.5% at 30°C, 72.3% at 33°C, and 78% at 36°C compared with those of the control.

The CAT activities were significantly (*p* < 0.01) increased across all temperatures (27, 30, 33, and 36°C) and all durations (*p* < 0.001) (1, 3, 6, and 10 h) compared with control (25°C), and the interaction between temperature and duration was significant (*p* < 0.001). When the exposure lasted for 6 and 10 h, the CAT activities at 33°C reached 8.2 ± 0.1 μ mg^–1^ FW for 6 h exposure and 9.3 ± 0.2 μ mg^–1^ FW for 10 h exposure, whereas the highest activity was found at 36°C for exposure of 1, 3, 6, and 10 h (5.2 ± 0.2 μ mg^–1^ FW for 1 h, 7.2 ± 0.3 μ mg^–1^ FW for 3 h, 8.3 ± 0.4 μ mg^–1^ FW for 6 h, and 10.4 ± 0.3 μ mg^–1^ FW for 10 h) (Figure 1C).

### Effect of Heat Stress on H_2_O_2_ and Malondialdehyde Contents in *Myzus persicae*

**FIGURE 2:**
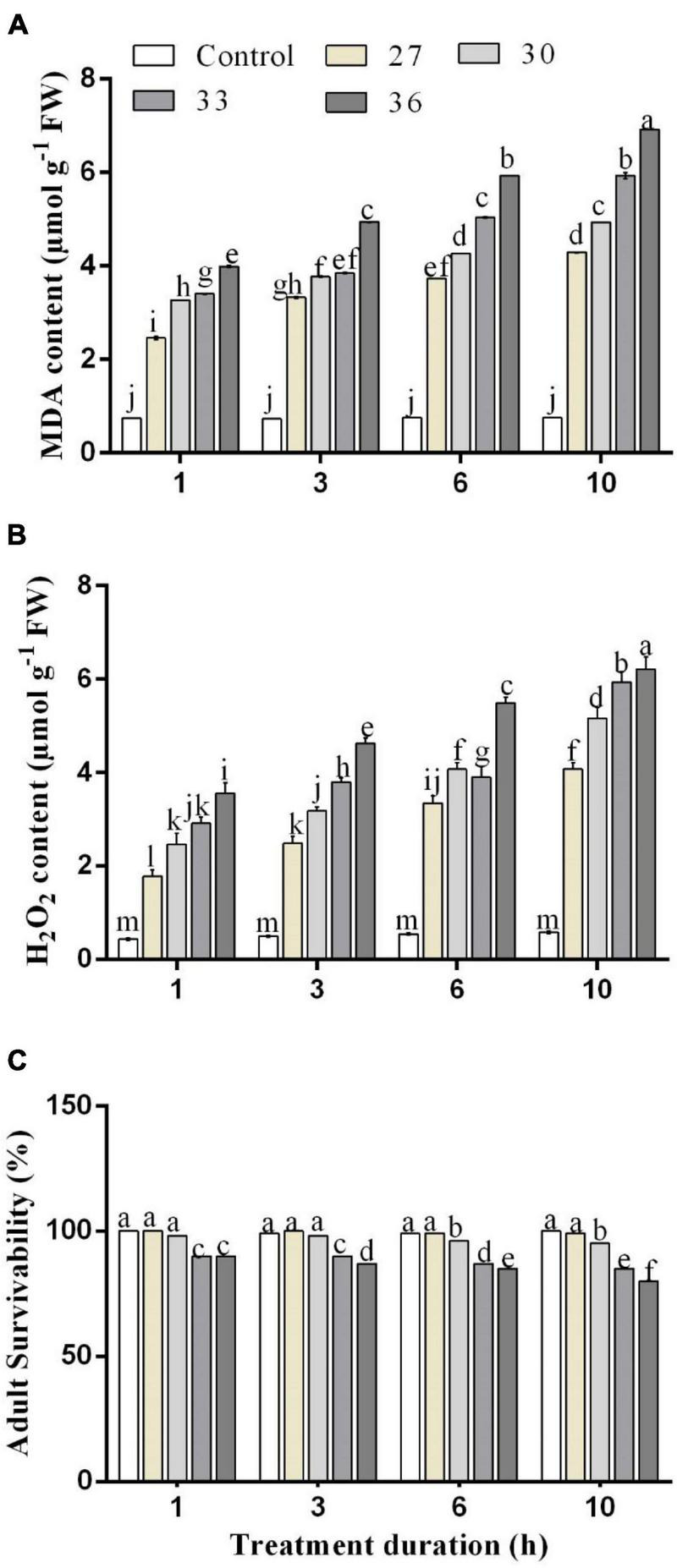
Changes in **(A)** malondialdehyde (MDA) and **(B)** hydrogen peroxide (H_2_O_2_) contents. **(C)** Effect of heat stress on survivability in *M. persicae* under different heat stresses (27, 30, 33, and 36°C) at different durations (1, 3, 6, and 10 h) in growth chambers. Small bars represent the SE of the means. Different lower case letters indicate significant differences between treatments at Tukey’s HSD test (*p* < 0.05) using one-way ANOVA.

The influence of different temperatures on MDA and H_2_O_2_ contents in *M. persicae* is presented in Figure 2. The MDA and H_2_O_2_ contents in *M. persicae* adults were significantly (*p* < 0.01) affected by all heat shock treatments (27, 30, 33, and 36°C), and all exposure times (*p* < 0.01) (1, 3, 6, and 10 h) compared with control and temperature and duration interacted significantly (*p* < 0.01). The results showed that the highest content of MDA was formed at 36°C for 1, 3, 6, and 10 h (3.7 ± 0.14 μ mg^–1^ FW for 1 h, 4.9 ± 0.1 μ mg^–1^ FW for 3 h, 5.9 ± 0.2 μ mg^–1^ FW for 6 h, and 7.1 ± 0.2 μ mg^–1^ FW for 10 h). Even for 1 h exposure time, the content of MDA increased considerably with increasing temperatures. The H_2_O_2_ content was found to have a significant difference among the different temperatures and exposure times. The highest content of H_2_O_2_ was formed at 36°C for 6 and 10 h (5.4 ± 0.3 μ mg^–1^ FW for 6 h and 6.2 ± 0.2 μ mg^–1^ FW for 10 h).

### Expression of Heat-Shock Proteins in *Myzus persicae* Response to Heat Stress

**FIGURE 3:**
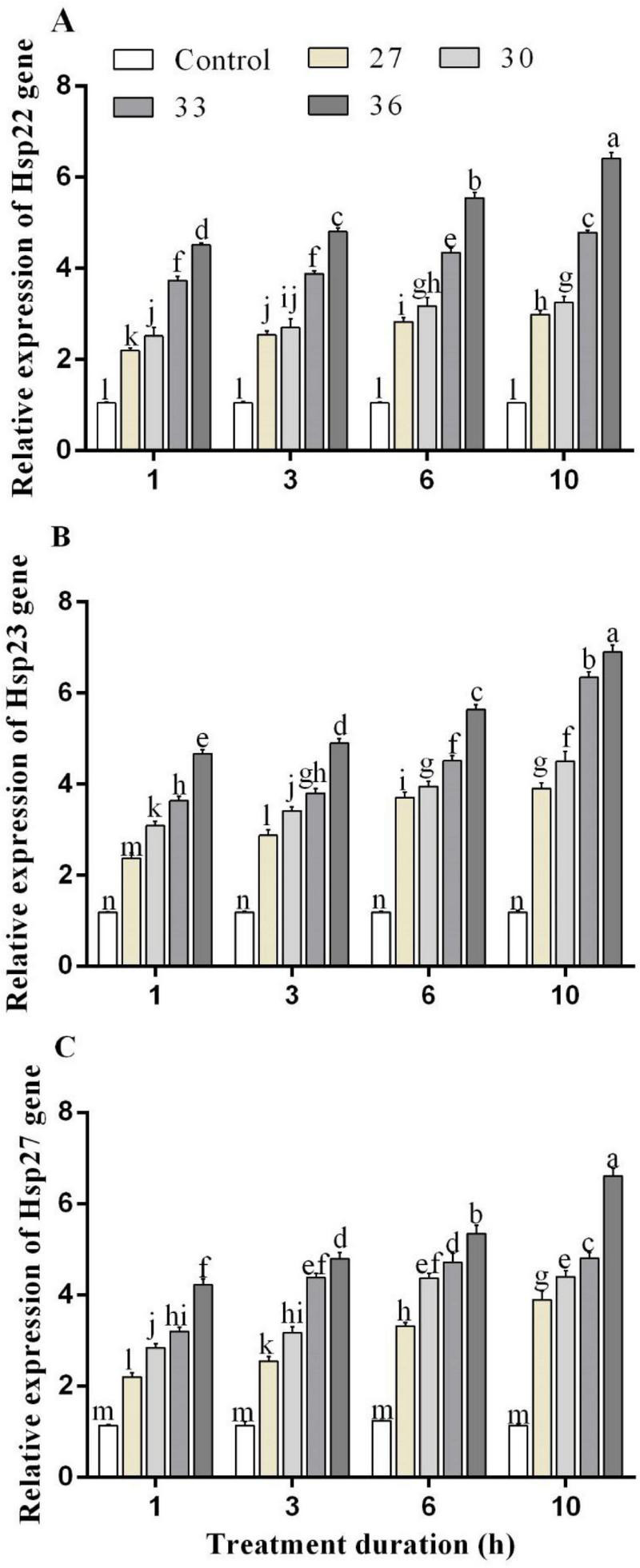
Relative expression of **(A)** Hsp 22, **(B)** Hsp 23, and **(C)** Hsp 27 genes in *M. persicae* under different heat stresses (27, 30, 33, and 36°C) at different durations (1, 3, 6, and 10 h) in growth chambers. Small bars represent the standard errors of the means. Different lower case letters indicate significant differences between treatments at Tukey’s HSD test (*p* < 0.05) using one-way ANOVA.

The RT-qPCR experiment was performed to analyze the expression profiles of HSPs genes in response to heat stress. Under short-time heat stress, the expression of *Hsp22*, *Hsp23*, and *Hsp27* increased. The *Hsp22* expression levels in *M. persicae* were significantly induced by heat stress at 36°C for 1, 3, 6, and 10 h exposure times and upregulated by 4.5, 4.8, 5.5, and 6.4-folds, compared with those of the control. *Hsp23* expression level increased by all heat treatments and highly increased at 36°C for 6 and 10 h by 5.6 and 7.0-folds. Exposure at 36°C for 6 and 10 h increased *Hsp27* expression level by 5.3 and 6.6-folds, respectively, compared with control (Figures 3A–C). In response to high-temperature treatments (27, 30, 33, and 36°C for 10 h), *Hsp23* transcript levels increased by 3.9, 4.5, 6.3, and 6.9-folds, more than the transcript levels of *Hsp22* by 2.9, 3.2, 4.7, and 6.4-folds and *Hsp27* by 3.8, 4.4, 4.8, and 6.6-folds.

### Expression of Antioxidant Enzyme Genes in *Myzus persicae* Response to Heat Stress

**FIGURE 4:**
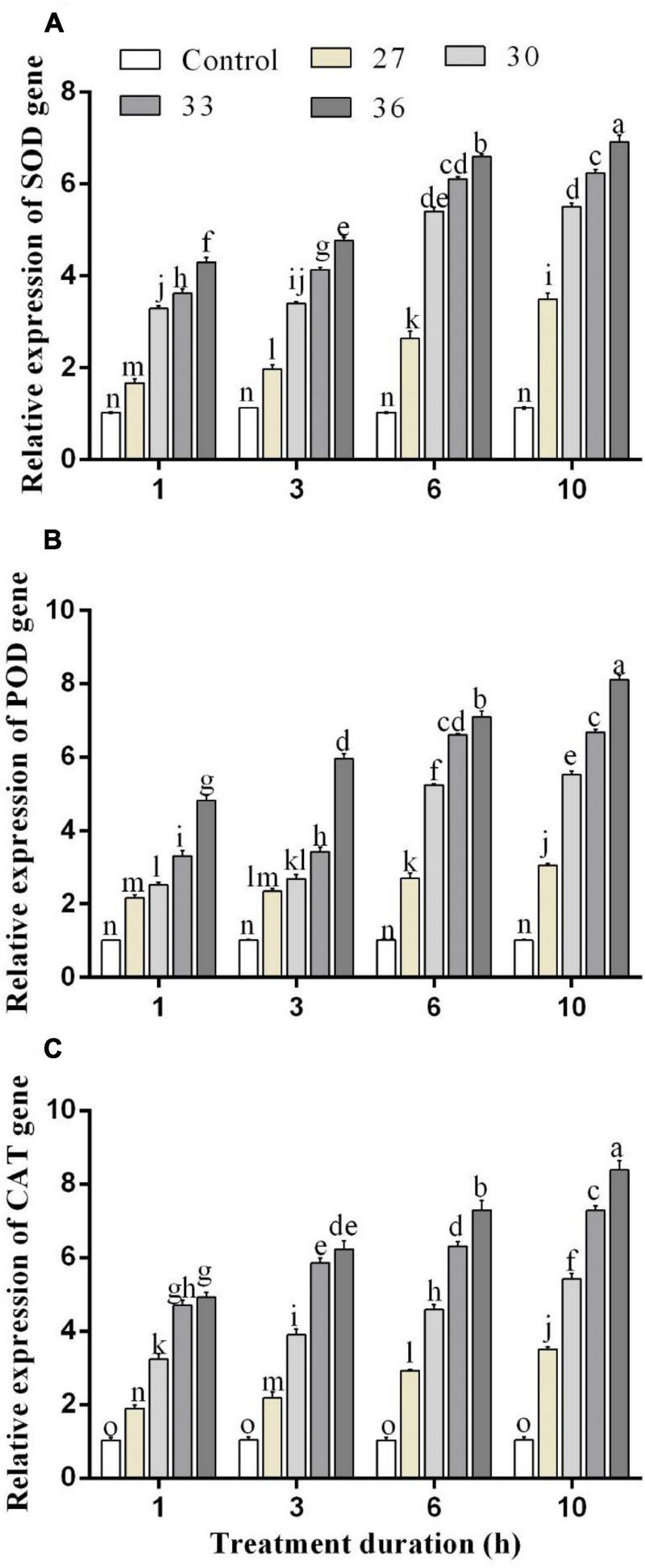
Relative expression of **(A)** SOD, **(B)** POD, and **(C)** CAT genes in *M. persicae* under different heat stresses (27, 30, 33, and 36°C) at different durations (1, 3, 6, and 10 h) in growth chambers. Small bars represent the standard errors of the means. Different lower case letters indicate significant differences between treatments at Tukey’s HSD test (*p* < 0.05) using one-way ANOVA.

The expressions of antioxidant enzyme genes were significantly (*p* < 0.05) increased by short-time heat stress. In all heat shock treatments (27, 30, 33, and 36°C) for four exposure times (1, 3, 6, and 10 h), the expression of *SOD*, *POD*, and *CAT* genes was increased but highly increased at 36°C. Exposure to high temperatures for 1, 3, 6, and 10 h significantly increased the expression level of the *SOD* gene by 4.2, 4.7, 6.6, and 6.9-folds in comparison with control, respectively (Figure 4A). The levels of the *POD* gene expression were upregulated at 36°C for 1, 3, 6, and 10 h by 4.8, 6.2, 7.0, and 8.1-folds, and similarly, the expression level of the *CAT* gene highly increased at 36°C for 6 and 10 h by 7.4 and 8.6-folds compared with control, respectively (Figures 4B,C). While at all temperatures (27, 30, 33, and 36°C for 10 h), the expression was induced by 3.5, 5.6, 7.4, and 8.6-folds for the *CAT* gene, by 3.4, 5.5, 6.2, and 6.9-folds for the *SOD* gene, and 3.0, 5.5, 6.6, and 8.1-folds for the *POD* gene.

### *Myzus persicae* Survivability Under Short-Time Heat Stress

**FIGURE 5:**
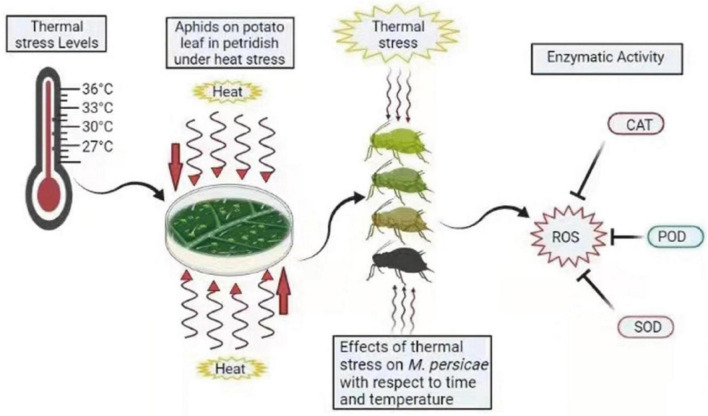
Summary diagram of antioxidant enzymes in *M. persicae* under thermal stress. Different colors of aphids indicate the effect of heat stress on aphids. ROS, reactive oxygen species; CAT, catalase; POD, peroxidases; SOD, superoxide dismutase. The zigzag arrows indicate the heat waves affecting aphids.

An increase in temperature combined with stress duration significantly (*p* < 0.01) decreased the survival rate of the aphids (Figures 2C, 5). Across the four temperature treatments (27, 30, 33, and 36°C), the survival rate of the aphids treated at 10 h decreased by 0.60, 4.04, 9.26, and 12.27%, respectively, compared with control (25°C).

## Discussion

This study reports the physiological and molecular response to short-time heat stress in *M. persicae*. It confirms that the antioxidant enzyme activity of SOD, POD, and CAT and their transcript levels were upregulated by increasing temperatures relative to those at 25°C. This is in agreement with many studies that high temperatures stimulate the defense mechanisms of insects, such as antioxidants and HSPs (Yang et al., 2010; Kang et al., 2017). In this study, our results showed that in all heat shock treatments (27, 30, 33, and 36°C) for four exposure times (1, 3, 6, and 10 h), the expression of *SOD*, *POD*, and *CAT* genes was increased compared with control.

Our research showed that the activity of antioxidant enzymes in *M. persicae* depends on temperature. We hypothesized that to retain physiological homeostasis, antioxidant enzymes scavenge the ROS generated by thermal stress. The SOD plays an important role in protection against ROS and reducing superoxide radicals produced by high temperatures (Park et al., 2009). An increase in SOD activity is the first stage of aphid response to oxidative stress. The SOD activity of *M. persicae* can be the adaptive reaction to reduce superoxide anion toxicity caused by high temperatures, as shown in *Panonychus citri* and *Propylaea japonica* (Yang et al., 2010; Zhang et al., 2015). CAT is the major H_2_O_2_ scavenging enzyme and works together with SOD to slowly reduce oxygen (Cui et al., 2011). In this study, the activity of CAT increased in *M. persicae* under different heat stress (27, 30, 33, and 36°C) compared with control (25°C). The increase in exposure times (1, 3, 6, and 10 h) equally increased CAT activities at 36°C. Previous studies by Dampc et al. (2020) discovered the effects on the activity of enzymatic markers in aphid tissues. *A. pomi* defense responses varied depending on the temperature at 28°C and survived due to flexible enzyme activity. Similarly, in this study, both the SOD and CAT enzymes in *M. persicae* tissues increased as the temperature and the stress duration increased, which contributes to their survivability. Studies on *Mythimna separata* revealed that increasing temperatures beyond the optimal conditions (25°C), increased SOD and CAT activities (Ali et al., 2017). POD oxidizes phenols and other aromatic compounds with the participation of H_2_O_2_. This group of enzymes plays an important role in the oxidation of secondary metabolites, which greatly facilitates feeding by insects (Urbanska et al., 1998).

In this study, the MDA content increased across all treatments, and a significant increase was observed at 36°C for 3, 6, and 10 h. The increase in H_2_O_2_ content at 36°C for 10 h was slightly different from the MDA content. This shows that MDA is an oxidative stress biochemical indicator and a major oxidant of peroxidized polyunsaturated fatty acids (Rael et al., 2004; Del Rio et al., 2005). Higher MDA levels were associated with increased electrolyte loss and H_2_O_2_ accumulation (Nandwal et al., 2007), which has long been used as a marker of stress tolerance due to LPO. The H_2_O_2_ content at 36°C for 10 h increased, but the MDA concentration significantly increased higher at 36°C for 10 h. These results indicate that the high-temperature stress in *M. persicae* resulted in increased lipid damage by ROS and may associate with LPO and other oxidative stress responses as reported in *P. japonica* and *Hylyphantes graminicola* (Zhang et al., 2015; Zhao et al., 2020).

The oxygen and capacity limitation of thermal tolerance (OCLTT) theory explains the first line of thermal limitation in animals at the whole-organism level and may represent an evolutionary constraint that was modified depending on life stage and climate, as well as during the transition to life in the air (Pörtner et al., 2017). Thermal acclimatization and adaptation change limits *via* modifying membrane composition, enzyme and mitochondrial capacity, or molecular integrity-protecting mechanisms (Pörtner, 2012). At thermal extremes, oxygen scarcity causes the transition to passive tolerance, associated systemic and cellular stress signals such as hormonal responses or oxidative stress, and the usage of defense mechanisms such as HSPs (Kassahn et al., 2009). In this study, increasing the temperature caused an oxygen shortage, which resulted in oxidative stress or the generation of ROS. As a result, the aphid’s performance capacity was mostly determined by biochemical mechanisms that established the aerobic capacity of cells and tissues in the aphids, specifically the capacity of ventilatory and circulatory organs to supply enough oxygen to pay physiological costs above maintenance. The notion of oxygen- and capacity-limited thermal tolerance (Pörtner and Knust, 2007; Pörtner and Farrell, 2008) describes how insufficient oxygen supply on both sides of the thermal window limits the aerobic scope and determines the performance window in animals (Pörtner and Knust, 2007). In this work, antioxidant activity and gene expression of SOD, CAT, and POD, as well as HSPs, such as Hsp22, Hsp23, and Hsp27, scavenge the ROS as an underlying mechanism of aphid thermal heat tolerance. According to a recent study by Pörtner (2010), a twist in the interpretation of existing knowledge is appropriate for determining the physiological aspects of the environmental niche in which a species or one of its specific life stages exists. The study of performance characters in connection to both biotic and abiotic characters results in such dimensions. As a result of recent insights into the mechanisms of thermal adaptation and limitation, the influence of temperature on terrestrial insects, such as aphids, has emerged as a unifying notion in the cause-and-effect understanding of climate change effects on terrestrial ectotherms.

Previous experiments have shown that HSPs play a significant role in insect thermal resistance (Parsell and Lindquist, 1993) and various environmental stresses, especially heat and cold stress (Kang et al., 2009; Hu et al., 2014). For example, the expressed levels of Hsp23 genes in female silverleaf whiteflies *Bemisia tabaci* were found to have a crucial role in heat tolerance at 44°C (Lü and Wan, 2011). In insects, forced production of HSP 27 (Hsp27) reverses drug efflux *via* P-glycoprotein (*ABCB1*) and *MDR1* gene expression (Zhao and Jones, 2012). Furthermore, the expression of the Hsp22 gene was raised in deep diapausing calanoid copepods like *Calanus finmarchicus* and played a function in both short-term stress responses and shielding proteins from degradation during diapause (Aruda et al., 2011). However, because Hsp22, 23, and 27 have never been shown to play a function in aphid heat tolerance in the short term, we focused on determining their role in *M. persicae* heat stress tolerance.

In this work, we observed that under short-time heat stress (1, 3, 6, and 10 h), the expression of *Hsp22*, *Hsp23*, and *Hsp27* was increased. The expression of *Hsp22*, *Hsp23*, and *Hsp27* genes at 36°C for 10 h was significantly higher than the control at 25°C (without heat) and other temperatures. The gene expression of *Hsp27* has been greatly stimulated by thermal stress exposure at 35°C for 2 h in *Chironomus riparius* (Martínez-Paz et al., 2014). Many experiments have shown that the *Hsp23* gene played a vital role in heat tolerance, and insects with higher *Hsp* gene expression had a higher survival rate under heat shock tolerance (Zhao and Jones, 2012). The expression of *Hsp23* was significantly increased at 36°C as the time exposure increased (1, 3, 6, and 10 h). Our results show that *Hsp22*, *Hsp23*, and *Hsps27* can greatly assist *M. persicae* in tolerating heat and thus contribute to the evolution of heat tolerance in this pest because *Hsp* gene expression increased at all temperatures; however, at 36°C for 10 h, *Hsp* gene expression increased more than other temperatures. These findings indicate that when *Hsp22*, *Hsp23*, and *Hsp27* levels rise at high temperatures, these genes can support *M. persicae* in developing heat tolerance. According to our findings, HSPs and antioxidant enzymes work together to defend against thermal damage and are implicated in the antioxidant response to thermal stress in *M. persicae* adults, allowing them to adequately respond with ROS caused by thermal stress.

## Conclusion

In this study, potential mechanisms of antioxidant reaction and HSPs in *M. persicae* were investigated. According to our knowledge, the physiological and molecular response in *M. persicae* with different temperatures and exposure times is reported for the first time by this study. According to our findings, short-time heat stress disrupts the function of redox in *M. persicae*. When the balance of redox processes is disrupted by external influences, oxidative stress develops. The main proposed factor for inducing oxidative stress in *M. persicae* is heat stress. In the antioxidant reaction to heat stress, antioxidant enzymes, such as SOD, CAT, and POD, as well as HSPs, such as Hsp22, Hsp23, and Hsp27, may be involved in the management of oxidative damage caused by MDA and H_2_O_2_, thus ROS. Aphids may respond rapidly to environmental stress due to the effective activation of enzymes, which allows them for environmental adaptation. Furthermore, we suggest more studies on *M. persicae* tolerance to heat stress involving the underlying mechanisms of oxidative stress tolerance.

## Data Availability Statement

The raw data supporting the conclusions of this article will be made available by the authors, without undue reservation.

## Author Contributions

AK and RI contributed to the study concept and design, and statistical analysis. AK, RI, IU, and SB contributed to the analysis and interpretation of data. CL and AT contributed to the investigation and resources. AK contributed to the drafting of the manuscript. J-JZ contributed to the review, editing, and proofreading of this manuscript. CZL contributed to funding acquisition and study supervision. All authors have read and agreed to this manuscript.

## Conflict of Interest

The authors declare that the research was conducted in the absence of any commercial or financial relationships that could be construed as a potential conflict of interest.

## Publisher’s Note

All claims expressed in this article are solely those of the authors and do not necessarily represent those of their affiliated organizations, or those of the publisher, the editors and the reviewers. Any product that may be evaluated in this article, or claim that may be made by its manufacturer, is not guaranteed or endorsed by the publisher.

## Supplementary Material

Supplementary Figure 1 Results obtained by 1% agarose gel electrophoresis of *M. persicae* RNA under different heat stresses (25, 27, 30, 33, and 36C), where M represents the marker.
