# Supplementary figures and images for: Antioxidant Enzymes and Heat-Shock Protein Genes of Green Peach Aphid (Myzus persicae) Under Short-Time Heat Stress
Source: Front Physiol. 2021 Dec 17;12:805509. doi: 10.3389/fphys.2021.805509 (PMC8718642; doi:10.3389/fphys.2021.805509)

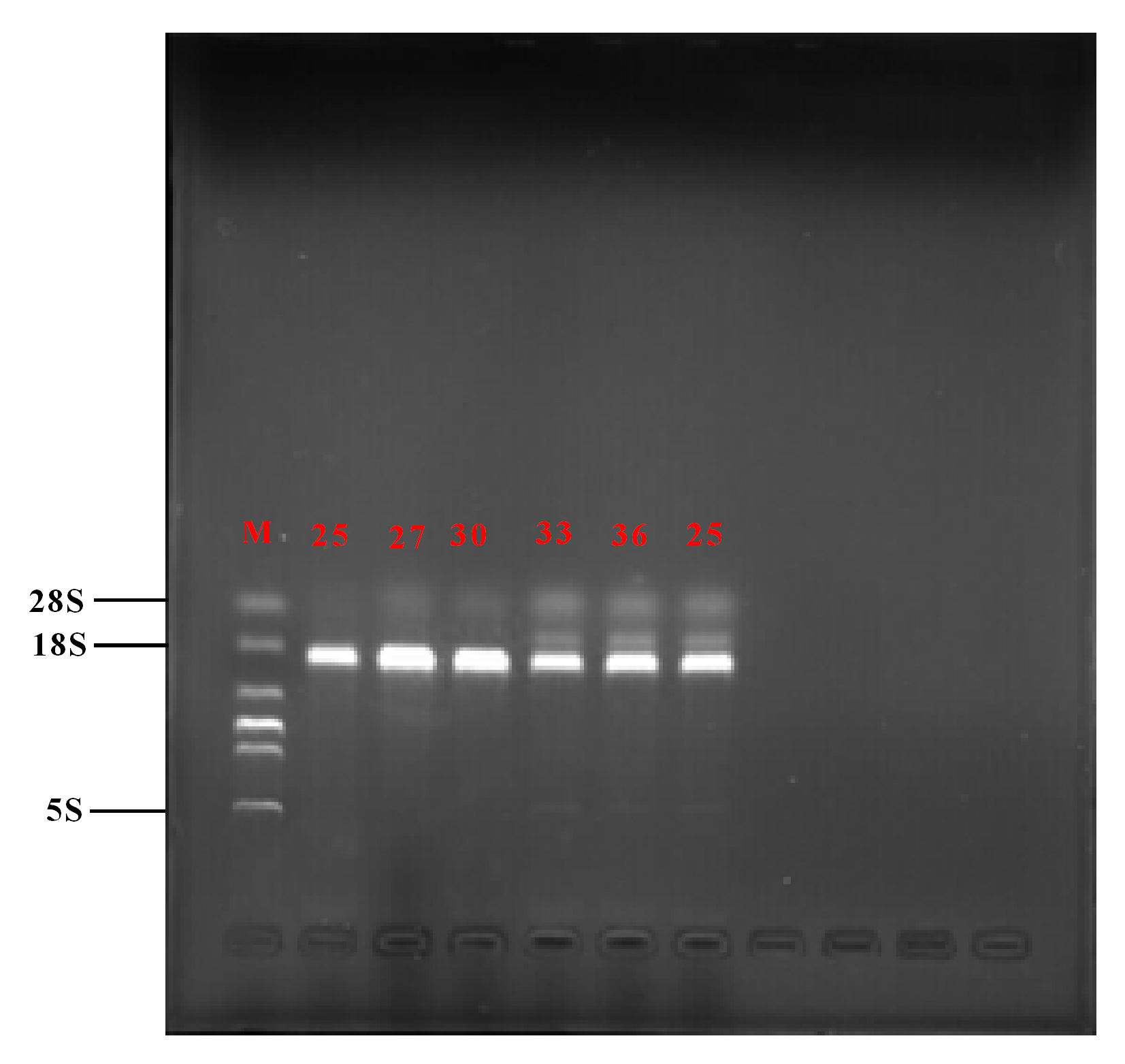

Supplement: Supplementary Figure 1 — Results obtained by 1% agarose gel electrophoresis of M. persicae RNA under different heat stresses (25, 27, 30, 33, and 36C), where M represents the marker. [file Image_1.JPEG]
