# Supplementary material for: Antioxidant Enzymes and Heat-Shock Protein Genes of Green Peach Aphid (Myzus persicae) Under Short-Time Heat Stress
Source: Front Physiol. 2021 Dec 17;12:805509. doi: 10.3389/fphys.2021.805509 (PMC8718642; doi:10.3389/fphys.2021.805509)
Supplement: Supplementary file 2 [file Table_1.DOCX]

Table 1. Primers used in the current study

| Primers | Primers Forward | Primers Reverse | PCR type |
| --- | --- | --- | --- |
| HSP22 | TTCGTGCTCCGCCCCATAAC | GGAAGAGCTCCAGCAGACGG | RT-qPCR |
| HSP23 | TGGCTTCCAGGTCTGCATGG | GGTGGAGGGCAACCATGAGG | RT-qPCR |
| HSP27 | CCCAAGGGCTTTGACCCCAA | GTCGGAGCGCATTGTCCAGA | RT-qPCR |
| SOD | GAAGAACCTCAAGCCTATCAGCG | CAGAGGGTGCTTTACAAGGATCT | RT-qPCR |
| POD | GCCGTTGAGATTACTGGTGGAC | GTCTTCCTGATGCTACCAAGGG | RT-qPCR |
| CAT | GAGGAAGCTATCAGAGTTGGAGGA | GCTCCTAGAGCTGTATTCCCAAGT | RT-qPCR |
| ACTIN | CAGTCGAAACGTGGTATCTTGACT | CGCCCAAGGACATCCTGATT | RT-qPCR |
